# Improving interoceptive ability through the practice of power posing: A pilot study

**DOI:** 10.1371/journal.pone.0211453

**Published:** 2019-02-07

**Authors:** Felicitas Weineck, Matthias Messner, Gernot Hauke, Olga Pollatos

**Affiliations:** 1 Department of Clinical and Health Psychology, Ulm University, Ulm, Germany; 2 Embodiment Resources Academy (ERA), Munich, Germany; Anglia Ruskin University, UNITED KINGDOM

## Abstract

Interoception refers to the detection and perception of signals from the inner body. Deficits in this domain have been linked to psychopathologies, prompting the search for strategies to improve this ability. Preliminary studies have shown that interoception could be enhanced through the manipulation of subjective feelings of power. We tested the effects of adopting powerful postures on different facets of interoception. Firstly, we measured the impact of a single power posing session on interoceptive ability in 41 healthy females. Then, the same participants were randomly assigned to two conditions (daily power posing practice vs. no practice). After one week the conditions alternated. Interoceptive accuracy, measured by the heartbeat tracking task, interoceptive sensibility, measured by the Body Perception Questionnaire (BPQ) and confidence ratings, as well as subjective feelings of power were assessed at baseline, after a single power posing session and after one week of training. A single power posing session significantly increased individuals' interoceptive accuracy. Also, power posing reduced individuals' scores on the BPQ after one week of daily practice and increased subjective feelings of power after one session and one week of daily practice. These findings suggest that adopting powerful postures has the potential to increase interoceptive accuracy, as well as subjective feelings of power, and to reduce interoceptive sensibility, measured by questionnaire.

## Introduction

Interoception refers to the perception of internal bodily sensations [1]. There is growing evidence supporting its crucial role in domains such as body ownership and selfhood [2, 3], intuitive judgment and decision-making [4, 5], emotional experience [6–8], emotional processing [9–11], behavioral self-regulation [12] and body image [13, 14]. Also, several studies targeting clinical populations have shown that deficits in the different interoceptive dimensions seem to be related to various psychopathologies [15]. Low interoceptive accuracy (IAcc) has, for example, been linked to major depression [16–18] and anorexia nervosa [19–21], whereas heightened IAcc appears to be prominent in anxiety disorders [22–24]. Considering the role of interoception in mental health further, a study by Füstös et al. [25] found that when using cognitive reappraisal as an emotion-regulation strategy, interoceptive accuracy facilitated the down-regulation of negative affect. In keeping with this observation, Kever et al. [26] also showed that individuals with higher accuracy made more use of reappraisal and suppression strategies, when regulating their emotions, compared to individuals with lower interoceptive accuracy. These findings imply that the awareness of ongoing bodily processes fosters the response to affect-related arousal.

Despite advances in our understanding of the different dimensions and effects of interoception, as well as research highlighting interoceptive deficits in clinical populations, little is known about effective strategies of *improving* this ability [27]. Evidence for possible interventions that could enhance interoceptive skills comes from studies showing that particular populations display increased interoceptive abilities. For example, a recent study by Christensen et al. [28] found experienced ballet dancers to have better interoceptive accuracy than non-dancers. The authors explained their findings with a dual action model, which highlights the interplay of eliciting and attending to emotional states and expressing these immediately through the body (via dance). Another study by Schirmer-Mokwa et al. [29] found that professional musicians displayed better interoceptive accuracy than non-musicians. The researchers highlighted the role of enhanced multisensory integration, which is especially trained in professional musicians, as a possible mechanism of enhancing interoceptive skills. Evidence for increased interoceptive ability due to meditation practice remains mixed. On the one hand, there is a line of studies that could not find better interoceptive accuracy in experienced meditators [30, 31]. On the other hand, there is evidence that meditators indicate higher confidence in their interoceptive ability than non-meditators [32]. Moreover, it seems that training non-meditators in a body scan intervention for eight weeks can enhance their interoceptive accuracy [33].

Furthermore, there is preliminary evidence suggesting that interoceptive deficits can be targeted through the enhancement of *self-focus* [34]. Ainley et al. [35, 36], for example, found that interoceptive accuracy could be increased when individuals looked at themselves in a mirror or processed self-narrative information. However, this strategy does not appear to be effective for every population. Pollatos et al. [37], for example, highlighted that individuals suffering from anorexia nervosa do not seem to benefit from this method of interoceptive enhancement, as they might find it difficult to engage in self-confrontation. This prompts the question whether there are other pathways of inducing self-focus.

One alternative strategy of enhancing self-focus could be through the manipulation of a person's subjective feelings of *power* [34]. From an evolutionary perspective, powerful individuals are less dependent upon others for personal resources and are therefore less likely to divert their attention outwards towards those less dominant [38, 39]. Less powerful individuals, on the other hand, must pay careful attention to their environment to avoid threat and therefore have less capacity to divert their attention inwards [40, 41]. Thus, power appears to serve as a stress buffer that allows individuals to engage in self-focus [39]. In support of this theory, research has shown that individuals who are primed with high power take on a more self-oriented view and find it more difficult to engage in other-oriented perspectives than individuals who are primed with low power [42]. In comparison to powerless individuals, the powerful also appear to be less distracted by external information and report trusting their 'gut feeling' when it comes to decision making [43].

There are, to date, two studies, which looked at the effects of power manipulation on interoception per se. Kunstman and colleagues [44] investigated the effects of power priming on interoceptive accuracy and found that power priming increased IAcc in those with high levels of body dysmorphic symptomatology. In their study, power was manipulated via a word search puzzle containing high power words, as well as an experiential writing prime, where individuals were asked to remember a time when they had influence over others. In another recent study, Moeini-Jazani et al. [34] showed that inducing power through a powerful role (i.e. impersonating a manager in a manager-subordinate role playing task) positively impacted upon individuals' interoceptive accuracy.

The present study will extend the previous findings by using physical postures reflecting power (i.e. power-posing [45]) as a method of power manipulation, instead of solely relying on semantic or associative priming. This approach has several advantages. Firstly, power posing has repeatedly been found to increase subjective feelings of power (see [46] for a review). Furthermore, inducing power via an embodiment intervention is beneficial, as powerful postures have been shown to have stronger effects on the activation of implicit power than powerful roles [47]. Also, the application of powerful postures is a non-verbal, time-effective method, which can easily be applied without having to activate autobiographical memory or having to engage in prior role-play.

The presented study aims to explore the effects of a single session of power posing, as well as one week of daily practice, on the different dimensions of interoception, namely, interoceptive accuracy, sensibility, and awareness [24, 48]. *Interoceptive accuracy* reflects performance on behavioural tests of interoception, such as the heart beat tracking task (HBTT) [49]. *Interoceptive sensibility*, on the other hand, describes individuals' subjective beliefs about their interoceptive accuracy and is assessed with self-report measures, such as the Body Perception Questionnaire (BPQ) [50] or individuals' confidence ratings about their performance on the heart beat tracking task [1]. *Interoceptive awareness* refers to the concordance between interoceptive sensibility and accuracy [27]. By investigating effects on all three dimensions, one hopes to gain a more differentiated understanding of the potential benefits of power posing on interoceptive ability. In particular, we aim to explore which dimension is most likely to be affected by power posing. This is important, as patients with psychiatric disorders vary in regards to their interoceptive abilities. For example, Pollatos and Georgiou [51] found that women with bulimia nervosa showed no differences in interoceptive accuracy compared to healthy controls. They did, however, differ in terms of their interoceptive sensibility, measured by questionnaire. Exploring the effects on each dimension can potentially help to offer patients more tailored treatments in the future.

To address the research questions, participants underwent a single power posing session, as well as one week of daily power posing. Primary outcomes were measures of interoceptive ability, and secondary outcomes were measures of subjective feelings of power. Results were compared to baseline. It was hypothesised that (I) One session of power posing will have a significant impact on individuals' interoceptive accuracy, sensibility (confidence; BPQ) and awareness scores; (II) Daily power posing will have a significant practice effect on individuals' interoceptive accuracy, sensibility (confidence, BPQ) and awareness scores. (III) Power posing will have a significant effect on individuals' subjective feelings of power.

## Materials and methods

### Design

The first part of the study measured the effect of a single-session of power posing on interoceptive ability and subjective feelings of power. It had the form of a single group repeated-measures design. 42 healthy females were recruited at Ulm University. One participant was excluded from the study due to the diagnosis of major depression, leaving a total of 41 participants. The heartbeat tracking task (HBTT) (Schandry, 1981), a question on individuals' confidence on the task, as well as a fixed-format questionnaire, were administered before and after a single power posing intervention.

The second part of the study measured the effect of one week of daily power posing practice on interoceptive ability and subjective feelings of power. It had the form of a 2x2 crossover design. After the testing of the short-term effects in the laboratory, all 41 healthy females were randomly assigned to two groups by a lottery system. Group A underwent daily power posing practice for one week (one session in the morning and one in the evening), whilst Group B paused with the practice. After one week, Group A paused the power posing practice, and Group B practiced daily.

In addition to this study, we intended to investigate the effects of power posing in patients with eating disorders and planned to compare the data to the healthy sample. This was the reason for choosing a crossover design in the study reported here. In clinical trials, it ensures that every patient receives a form of treatment, and is not only part of a control group [52, 53]. It also offers higher power with a smaller sample size, which is particularly relevant as clinical participants can be difficult to recruit. Please note that the data of the clinical sample is not reported here.

### Participants

42 healthy Caucasian females were recruited from students at Ulm University. In return for their participation, they received course credit. They were informed that the study aimed to investigate the effects of physical postures on muscular strength. Participants had a mean age of 20.85 (*SD* = 1.84) and a mean BMI of 20.65 (*SD* = 2.29). Exclusion criteria were previous or present psychiatric or somatic disorders, age under 18 and specific experience with power posing. One participant was excluded from both parts of the study, as she was taking SSRIs and indicated suffering from depression, leaving a total of 41 participants. None of the remaining participants were taking medication (except contraceptives) or had a past or present psychiatric or severe somatic illness, as assessed by anamnestic questionnaire.

Group A and Group B did not differ significantly regarding their age (Group A: *M* = 20.81 (*SD* = 1.75); Group B: *M* = 20.90 (*SD* = 1.97); t(39) = - 0.156, p = .877) or level of fitness (Group A: *M* = 60.95 (*SD* = 11.03); Group B: *M* = 58.05 (*SD* = 21.90); t(28) = 0.532, p = 0.599).

### Materials

#### Questionnaires

An online questionnaire was used to collect health status and personal data one week before testing (including age, educational background, level of fitness, previous experience with body centred interventions). Level of fitness reflected the extent to which individuals felt physically fit. Participants could give an answer ranging from not at all (0) to very much (100). Questions on the experience with body-centred interventions asked individuals to indicate whether they practiced yoga, pilates, mindfulness, meditation or back training, to identify potential confounding variables. Participants could indicate their answer on a three-point scale ('never practiced'; 'practiced before, but not at the moment'; 'practising on a regular basis'). Also, different standardized psychological questionnaires were administered. The German version of the Patient Health Questionnaire (PHQ-D) was developed as a screening measure for mental disorders in primary care, based on the diagnostic criteria of DSM-IV [54]. In this study the long version of the questionnaire was used, that includes subscales screening for e.g. depression, somatoform symptoms, anxiety and psychosocial stress. All questions refer to the occurrence of symptoms over the past two to four weeks. Furthermore, the awareness subscale of the Body Perception Questionnaire [50] was administered. This measure has been previously used as a measure of interoceptive sensibility [1, 55]. The BPQ awareness subscale consists of 45 items reflecting bodily sensations. Examples of the items are 'I am aware of my eye movements' or 'I am aware of my mouth being dry '. Participants can indicate their awareness of each sensation on a five-point scale ranging from 'never' to 'always'. Also, the trait subscale of the state-trait anxiety inventory (STAI) was applied [56]. It is a validated measure that contains 20 items assessing individuals' trait anxiety on a four-point scale ranging from 'almost never' to 'almost' always (score range 20–80). At the end of the weekly power posing practice, participants were given an evaluative questionnaire constructed by the authors. They were asked how many times they practiced daily and could indicate their answers on a 4-point scale ('less than once per day'; 'once per day'; 'two times per day'; 'more than two times per day'). They were also asked whether they could fill in the diary as instructed. They could indicate their answers by choosing one of the following items 'yes, as instructed'; 'no, I had to make minor changes'; 'no, I had to make major changes'; 'no not at all'.

#### Measurements of interoceptive ability

**Interoceptive Accuracy (IAcc).** Interoceptive accuracy was assessed using the heart beat tracking task (HBTT) [49]. The task included four heartbeat counting trials using the mental tracking method. After a short training interval of 15s, four intervals of 35s, 45s, 25s and 60s followed. The four intervals were presented in a fixed order across participants. Participants were instructed to count their own heartbeats silently and to verbally report the number of counted heartbeats at the end of each counting phase. The beginning and the end of the counting intervals were indicated by a start and stop cue given by the experimenter. Participants were instructed not to take their own pulse or attempt to use any other form of manipulation to support the counting of their heartbeats. No prior information regarding the length of the counting phase was given. Participants received no feedback on the quality of their performance at the end of each trial. Heartbeat signal was recorded with the mobile heart frequency monitor RS800CX (Polar Electro Oy, Kempele, Finland). The RS800CX, records the inter-beat-interval, is non-invasive and can compete with other ECG measures regarding its validity and reliability [57, 58]. IAcc was calculated as the mean heartbeat perception score according to the following transformation:

$$\frac{1}{4}\sum (1-\frac{\left(\left|recorded\;heartbeats-counted\;heartbeats\right|\right)}{recorded\;heartbeats}$$

IAcc scores could range from 0 to 1. Higher scores indicated smaller differences between the counted and recorded heartbeat and thus better IAcc.

**Interoceptive Sensibility (IS).** Interoceptive sensibility is defined as an individual's self-confidence relative to his or her objective performance on the heartbeat tracking task (HBTT) [1]. After completion of the HBTT, participants were asked to rate their confidence in their performance on a scale from one to ten (1 = not confident at all; 10 = fully confident) and to verbally report their rating to the experimenter. As a secondary measure of interoceptive sensibility, the Body Perception Questionnaire [50] was applied.

**Interoceptive Awareness (IAw).** Interoceptive awareness refers to the concordance between interoceptive sensibility- and accuracy measures [27]. To operationalise interoceptive awareness, we applied the 'percent of maximum possible' or POMP scoring method described by Cohen et al. [59]. A POMP score is the result of a linear transformation of any raw metric into a 0 to 100 scale. For this study, interoceptive accuracy and confidence scores were converted into POMP scores to make them comparable. The absolute difference between POMP scores was then calculated as a variable representing IAw. For the scaling of the awareness score to be intuitive, i.e. for higher scores to represent higher awareness, the calculated absolute difference between POMP scores was again subtracted from 100. This resulted in an awareness score between 0 and 100. This approach has the benefit, that an individual awareness score, reflecting the correspondence between interoceptive accuracy and confidence, could be generated for each participant. In addition to this approach, the pearson correlation *r*, between accuracy and confidence was used as an index of interoceptive awareness [60]. A stronger correlation, after power posing, would suggest that interoceptive awareness has improved relative to baseline. For this calculation, the data was collapsed after training or after pause respectively.

#### Measurements of subjective feelings of power

Several power measures were included as a manipulation check. We thereby assessed subjective feelings of power, as well as *explicit*, and *implicit* power motives. Explicit motives reflect aspects of the self-concept that the individual is consciously aware of [61]. Implicit motives, on the other hand, are not consciously represented but still guide and select spontaneous behaviour [62]. Previous research has highlighted that explicit and implicit motives often diverge from one another [63–65]. This observation prompted us to choose a multi-dimensional approach to determine subjective sense of power.

To assess individuals' *explicit* feelings of personal power, we created a 6-item Likert scale in German, that was based on the Personal Sense of Power Scale by Anderson et al. [66] (item one to six). We refer to it as the Sense of Power Scale 6 German Version (SOPS 6 GV). The Cronbach's alpha of our scale was .815. Participants could indicate their agreement or disagreement on a six-point scale. The response options ranged from 1 = ‘strongly disagree’ to 6 = ‘definitely agree’. An example of a statement was 'I think I have a great deal of power'. Some of the statements were reverse scored to prevent response bias. Please refer to the supporting information section (S1 File and S2 File) for a full version of the scale.

Also, a visual analogue scale (VAS) was used, asking participants to indicate how powerful they felt, by marking their answer with a pen on the line. One end of the line was labelled with 0, indicating no power at all, whereas the other end was labelled with 100, indicating high power. To avoid participant's suspicion regarding the true nature of the study, the VAS of subjective feelings of power was embedded between two other visual analogue scales labelled 'energetic' and 'strong'. For the analysis, only the VAS power scale was used.

As a third measure of explicit power, the Self-Assessment Manekin Scale (SAM) was applied [67]. SAM measures felt pleasure, arousal and dominance through pictorial assessment. Participants are asked to circle one of five manekins that best reflected their current state on three scales. For the manipulation check, only the dominance subscale was used.

As an *implicit* measure of power, we used the Multi Motive Grid (MMG) by Sokolowski et al. [61]. The MMG is a semi-projective measure, assessing individuals' implicit motivation for affiliation, achievement and power. The measure contains a series of 14 drawings, depicting ambivalent social situations. Below every picture, a number of statements are given, and individuals are asked to judge whether each statement fits the given situation or not, by circling 'yes' or 'no'. The MMG distinguishes between hope and fear components of affiliation, achievement and power. In the case of power, the two components are 'hope for power' and 'fear of losing' power. Examples of power statements are 'anticipating to lose standing' or 'hoping to acquire a good standing'. The single motive scores for each picture are summarised to obtain a global score for each of the six motive components. Scores range from one to twelve. Previous studies have indicated good internal consistency and reliability of the measure [61, 68].

#### Power postures

**Fig 1:**
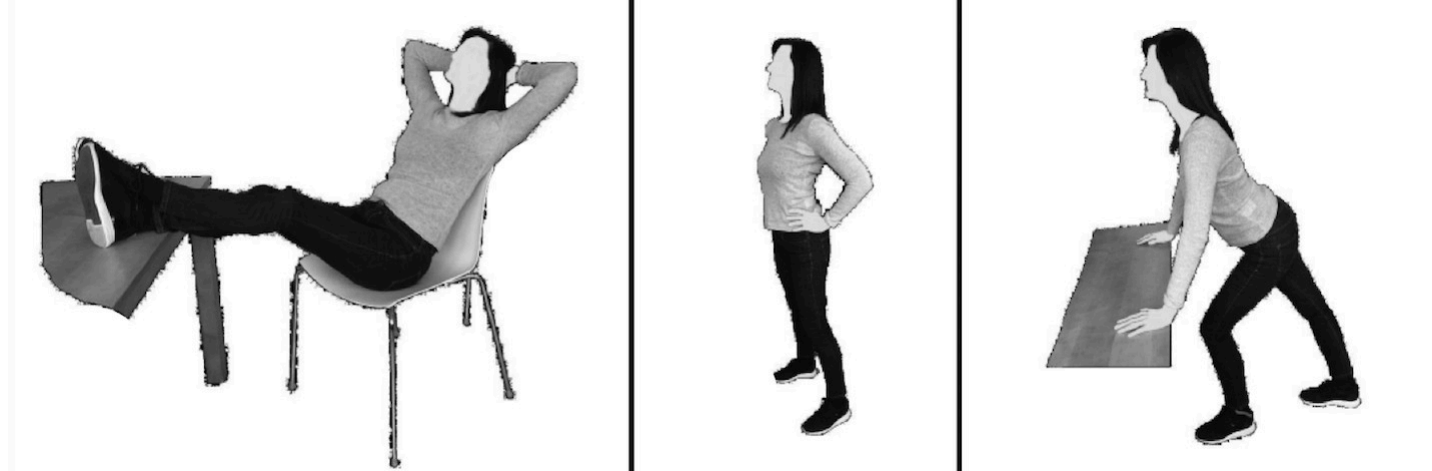
Power postures in the order of practice. Note. Postures were adopted in the presented order.

The bodily postures relevant for the training program were selected from previous research studies on non-verbal displays of power [45, 69, 70]. A total of three different power poses were applied. Each posture contained several elements associated with power such as, e.g. tilting the head up, leaning forward, placing the hands into the waist or adopting an open bodily position [71]. Please refer to Fig 1 for a full display of the power poses. The order in which the postures were adopted was fixed. Each pose was held for 45 seconds. This duration was chosen, based on previous studies showing beneficial effects after 60 to 120 seconds of power posing [46]. As we included three different poses, we reduced the previously used duration by 15 seconds per pose, as it has been proposed that holding the postures for too long might cause discomfort in the individuals (especially the feet-on-the-desk posture) and might lead to disadvantageous effects [46]. Thus, one session lasted for approximately six minutes, including short breaks between each posture to adjust the body. An audiotape, guiding individuals through each pose, was played during each session. The tape was recorded by a female experimenter and ensured that the poses were adopted correctly and homogenously. While adopting the poses, individuals were asked to fixate on a cross on the wall, placed one hand length above their individual height, at a 1.5 meter distance. Participants were also specifically instructed to engage in self-focus by continually shifting their attention between three anchor points (feet; sternum; chin), and to feel the space they were taking up. This self-focus component was added as we wanted to ensure that participants adopted the poses correctly when practising at home. Based on previous pilot testing, we realised that if attention is not paid to the three anchor points, it is likely that individuals will, for example, not push the sternum area outwards and quickly fall back into a slumped posture (especially in the feet-on-the-desk-posture). Due to a similar reason, the fixation cross was added. While developing the practice sequence of the power poses, we observed that the chin easily drops after a few seconds of holding the powerful posture, as holding the chin slightly upwards is not very natural and the body automatically falls back into its initial posture. Fixating a cross that directs the gaze of the participant upwards has the potential to control this aspect.

### Procedure

**Fig 2:**
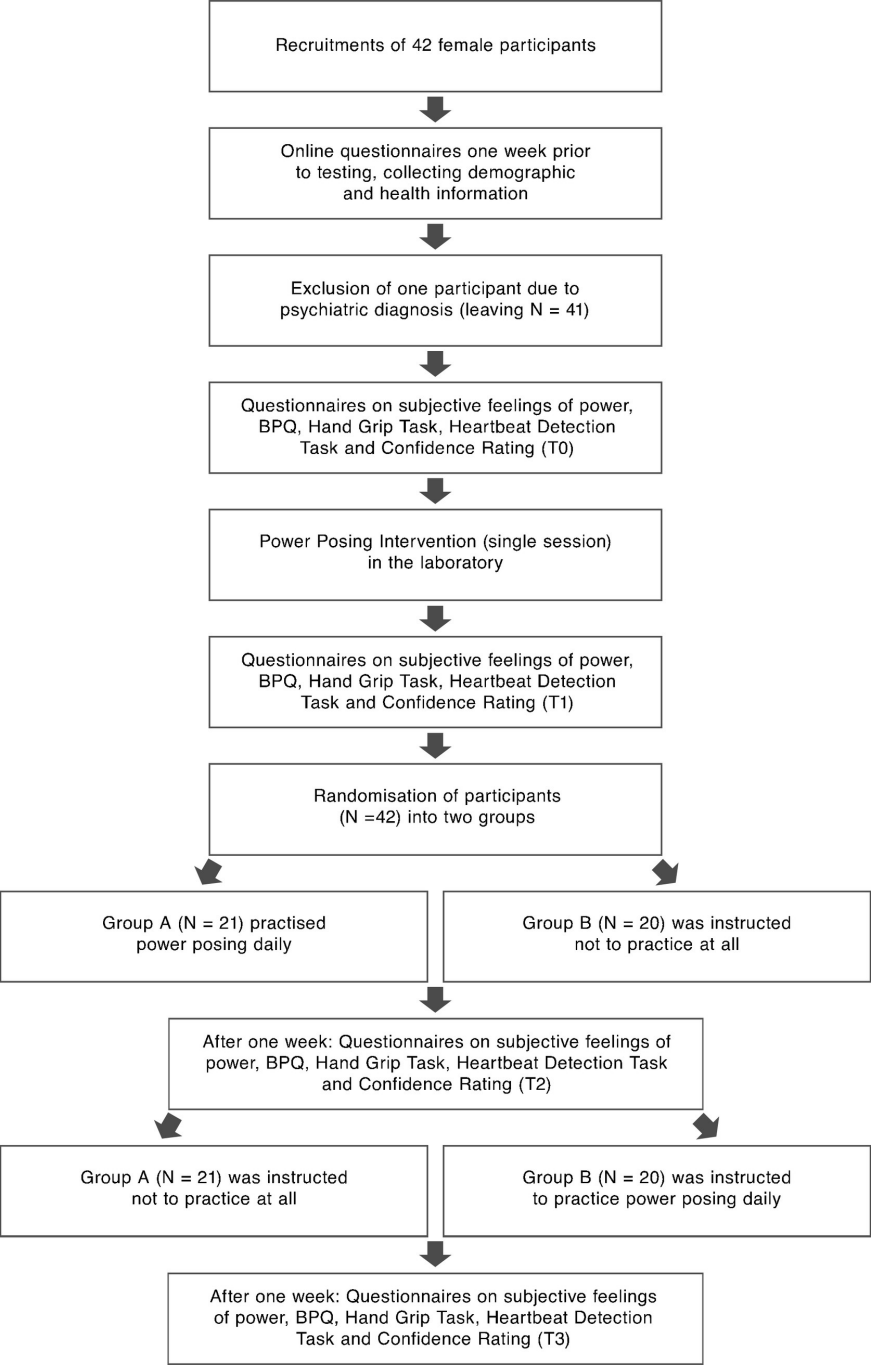
Flow chart outlining the procedure of the study and participants' experimental exposure over time.

The study was conducted in accordance with the Declaration of Helsinki, and ethical approval was obtained from the Institutional Review Board (IRB) of Ulm University. Before testing, informed consent was collected from the participants according to IRB requirements. Individuals were tested at the laboratories of the Clinical- and Health Psychology Department of Ulm University. They were informed that the study aimed to investigate the effects of physical postures on muscular strength to avoid power priming and response bias. To increase believability in the cover story, individuals were asked to count how many times they could push in a hand grip (HG). They were given 60 seconds to execute as many pushes with their dominant hand, as possible. Both sides of the HG clicking together counted as one push. The experimenter noted the number of final pushes on a separate sheet of paper. Furthermore, they completed the battery of questionnaires. Individuals' heartbeat perception was then tested at baseline (T0) and after a single power posing session (T1). The power poses were shown on a picture and subsequently presented in person by the experimenter. Individuals were then asked to adopt the pose themselves. After a short training session, that endured for as long as each participant needed to understand and apply the postures correctly (approximately one to two minutes), the audio instruction tape was played. During this procedure, the experimenter left the room to avoid reducing self-focussed attention due to social presence [72, 73]. After the testing of the one-session effects of power posing in the laboratory, participants were randomly assigned to two groups (A and B) via a lottery system. To avoid order effects, the training and the pause condition were counter-balanced between groups. Firstly, Group A was asked to practice the learnt postures daily (once in the morning and once in the evening) and Group B was explicitly told not to apply the poses again. To ensure that the poses were adopted correctly at home, participants were given the fixation cross, a printed picture of each pose, as well as the audio recording with the instructions. Heartbeat perception was tested after one week of power posing training or pause respectively (T2) and again after switching the experimental condition (T3). Participants filled in the questionnaires before and after the testing sessions. To increase compliance, individuals filled out a daily diary, noting the date and time of the power posing, as well as the poses they adopted. Please refer to Fig 2 for a flow chart outlining the procedure of the study and participants' experimental exposure over time. A diagram of all measures and intervention used is given in the supporting information section (S1 Table).

### Data analysis

All data were analysed using IBM SPSS Statistics 22 software (SPSS, Chicago, IL). Normality of the data was assessed using Shapiro Wilk test, Q-Q plot and histogram. The assumption of normality was upheld for all variables except BPQ and SOPS 6 GV due to outliers. A priori contrast analysis was performed using paired samples t-tests to investigate mean differences in interoceptive accuracy, interoceptive sensibility measured by confidence rating and the body perception questionnaire (BPQ), interoceptive awareness and subjective feelings of power (VAS; SAM), before and after a single power posing session (Baseline vs. T1). P value significance level cut-off was adjusted for multiple comparisons using the Holm-Bonferroni correction method. Interoceptive awareness was calculated by transferring interoceptive accuracy and confidence scores into POMP (percentage of maximum possible) scores, according to the method described by Cohen et al. [59]. Thereby the following formula was used: POMP = [(observed—minimum) / (maximum—minimum)] / 100. 'Observed' represented the observed score for a single case, 'minimum' reflected the minimum possible score on the scale, and 'maximum' reflected the maximum possible score on the scale. Then, the absolute difference between interoceptive accuracy- and sensibility POMP scores was calculated. At this stage, higher scores reflected higher discrepancies between interoceptive accuracy and confidence and thus, lower interceptive awareness. For the scaling of the awareness score to be intuitive, i.e. for higher scores to represent higher awareness, the calculated absolute difference between POMP scores was subtracted from 100, which resulted in an awareness score between 0 and 100. To investigate the effect of power posing after one week of training, data of the same outcome measures, collected after each power posing training period, was collapsed. It was ensured that there were no significant differences between the outcomes of power posing conditions before the data was collapsed. Missing data varied slightly between measures, and the numbers of cases are reported for each analysis.

## Results

### Demographics and questionnaire data

For a full list of demographic information, please refer to Table 1. Regarding compliance, all participants (N = 41) indicated that they had practiced power posing twice a day. All but one participant (97.6%) (N = 40), indicated that they had filled out the diary as instructed. Also, all participants, except one, (97.6%) (N = 40) stated that they had used the audiotape as instructed.

**Table 1:** Demographic information at baseline.

Variable	Baseline		
	*M*	*(SD)*	*[n]*
Age	20.85	(1.84)	[41]
Highest Education	5.00	(0.00)	[41]
BMI (kg/m^2^)	20.65	(2.29)	[41]
Level of Fitness	59.54	(17.05)	[41]
STAI	47.68	(4.74)	[41]

Note. M = Mean; SD = Standard Deviation; n = Number of Participants.

Score Ranges. Level of Fitness (0–100); STAI (20–80)

### Effects of a single session of power posing on interoceptive ability

Regarding the single session effect of power posing (please also see Table 2), participants displayed higher interoceptive accuracy after a single power posing session (*M* = 0.70; *SD* = 0.19), than before (*M* = 0.65; *SD* = 0.19). This difference, -0.04, BCa 95% CI [0.72, -0.01], was significant t(40) = -3.26, p = .002, and represented a small effect size, d = 0.23 (Cohen, 1988). Regarding interoceptive sensibility scores, the confidence rating of participants was not significantly higher after a single power posing session (*M* = 4.62; *SD* = 1.90), than before (*M* = 4.58; *SD* = 1.81); t(40) = -0.23, p = .819. Also, no significant difference was found in BPQ scores between baseline (M = 1.90; SD = 0.70) and T1 (M = 1.80; SD = 0.65); t(40) = 1.95, p = .059. BPQ scores did not significantly correlate with interoceptive accuracy at baseline (r = -.134; p = .405) or T1 (r = -.036; p = .821). Participants' interoceptive awareness score, was significantly lower after a single power posing session (*M* = 72.91; *SD* = 20.75) than before (*M* = 78.02; *SD* = 19.58), t(40) = 3.01, p = .005, indicating a higher discrepancy between interoceptive accuracy and confidence, after power posing, thus lower interoceptive awareness.

**Table 2:** Results of interoceptive measures.

	Measurement-Points			Test statistics			
	Baseline	After one session	After one week of training	Baseline to after one session		Baseline to after one week of training	
Variable	M (SD) [N]	M (SD) [N]	M (SD) [N]	T (df)	p	T (df)	p
BPQ	1.90 (0.70) [41]	1.80 (0.65) [41]	1.73 (0.65) [41]	t (40) = 1.95	.059	t (40) = 2.71	.010*
Interoceptive Accuracy	0.65 (0.19) [41]	0.70 (0.19) [41]	0.68 (0.18) [41]	t (40) = - 3.26	.002*	t (40) = -1.67	.103
Interoceptive Sensibility	4.58 (1.81) [41]	4.62 (1.90) [41]	4.77 (2.07) [41]	t (40) = - 0.23	.819	t (40) = -0.90	.375
Interoceptive Awareness	72.91 (20.75) [41]	78.02 (19.58) [41]	67.75 (21.40) [41]	t (40) = 3.01	.005*	t (40) = 2.20	.034

Note. M = Mean. SD = Standard Deviation. T = T-Test-Statistic. p = Significance Level.

* = Statistically significant difference after Holm-Bonferroni adjustment

For the analysis a two-tailed test was applied.

BPQ = Body Perception Questionnaire

Interoceptive Sensibility = Confidence in performance on HBTT task

Score Ranges. BPQ (1–5); IAc (0–1); IS (1–10); IAw (0–100)

The correlation between interoceptive accuracy and confidence was not significant at baseline (r = .304, p = .053) or at T1 (r = .305, p = .052).

**Fig 3:**
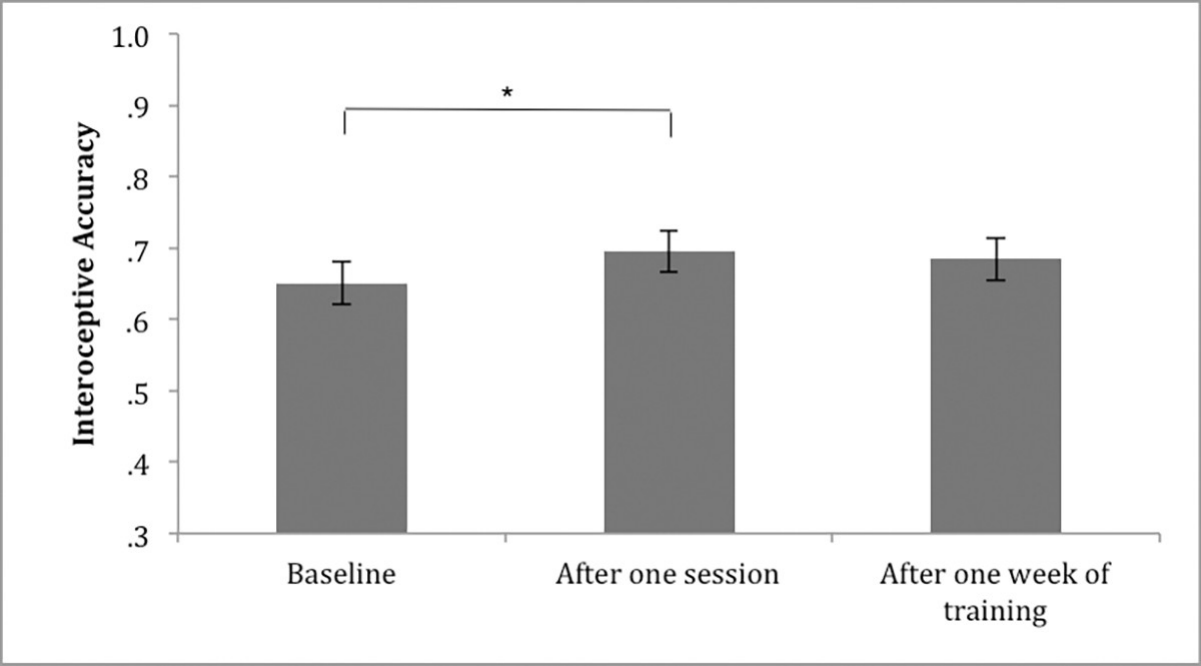
Interoceptive accuracy mean scores visualized by time point. Note. Error Bars represent SE.

Interoceptive accuracy mean scores sorted by time points (baseline; after a single, after one week of training) are visualised in Fig 3.

### Effects of one week of power posing on interoceptive ability

**Fig 4:**
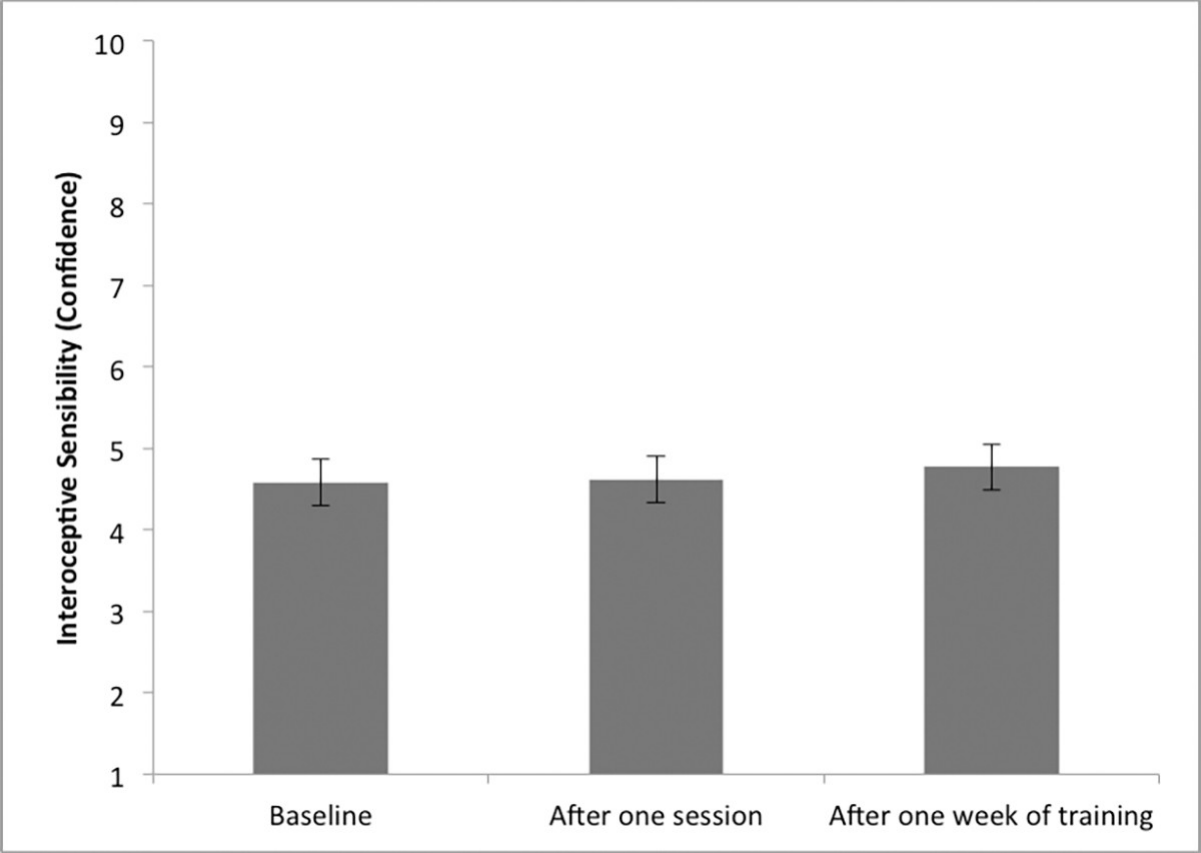
Interoceptive sensibility mean scores visualized by time point. Note. Error Bars represent SE.

**Fig 5:**
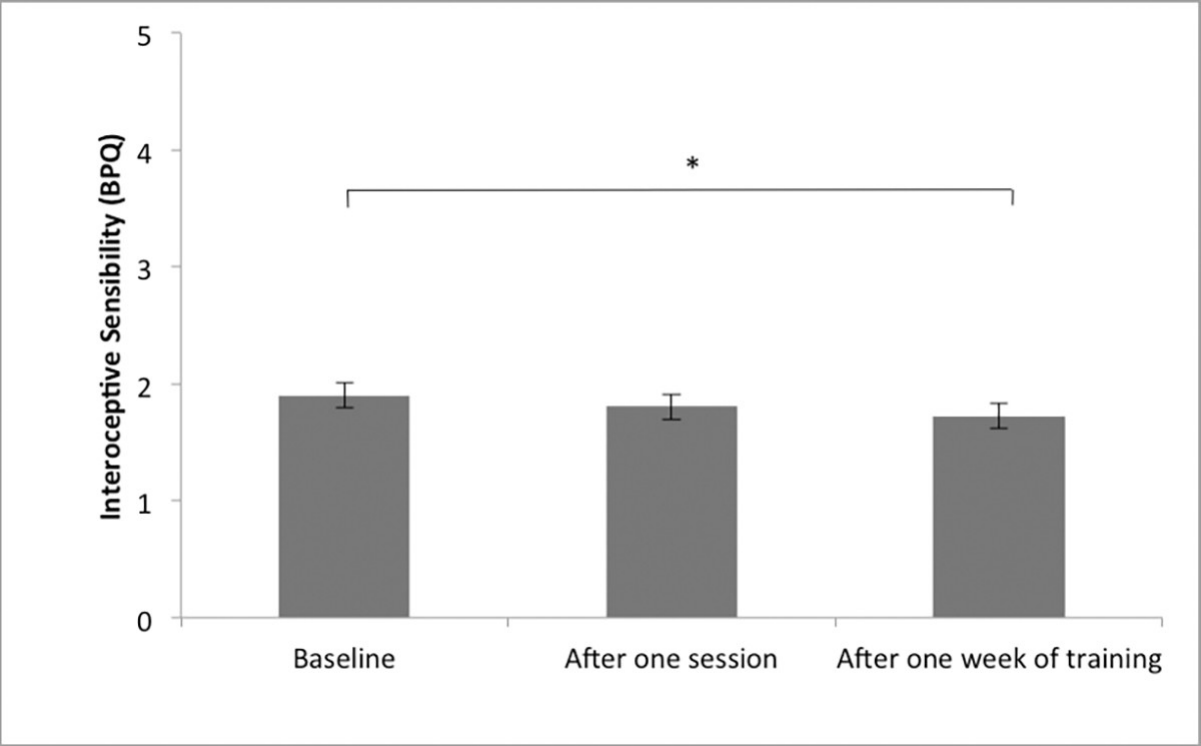
BPQ mean scores visualized by time point. Note. Error Bars represent SE.

Regarding the effect of one week of power posing, (please also see Table 2 and Figs 3–5), participants did not display higher interoceptive accuracy after one week of training (*M* = 0.68; *SD* = 0.18) compared to baseline (*M* = 0.65; *SD* = 0.19). t(40) = -1.670, p = .103. Regarding interoceptive sensibility scores, the confidence rating of participants was also not significantly higher after one week of training (*M* = 4.77; *SD* = 2.07) compared to baseline (*M* = 4.58; *SD* = 1.81), t(40) = -0.898, p = .375. When looking at interceptive sensibility with the BPQ measure, BPQ scores were significantly lower after one week of power posing training (*M* = 1.73; *SD* = 0.65) than at baseline (*M* = 1.90; *SD* = 0.70); t (40) = 2.71; p = 0.010. BPQ scores did not significantly correlate with interoceptive accuracy after training (r = .073; p = .652). Participants' interoceptive awareness score, was lower after one week of power posing (*M* = 71.93; *SD* = 18.38) than before (*M* = 77.40; *SD* = 18.19), t(40) = 2.76, p = .034, indicating a higher discrepancy between interoceptive accuracy and confidence after one week of power posing. However, this difference was no longer significant after Holm-Bonferroni correction was applied.

Interoceptive accuracy did not significantly correlate with confidence after one week of power posing practice (r = .045, p = .780) or practice pause (r = .220, p = .167).

### Subjective feelings of power

The internal consistency of the German 6-item sense of power scale (SOPS 6 GV) was assessed by Cronbach's alpha after the data was collected. Reverse scored items were recoded before the reliability analysis. Cronbach’s alpha was .815.

Paired samples t-tests were run to determine whether there was a statistically significant mean difference in VAS and SAM scores before and after a single power posing session. As can be seen from Table 3, power posing led to a significant increase in VAS scores, from Baseline (M = 39.78; SD = 19.15) to T1 (M = 47.80; SD = 20.09), t(40) = -4.754; p < .001. Participants did not show significantly higher SAM scores at T1 (*M* = 2.90; *SD* = 0.87) than at baseline (*M* = 2.65; *SD* = 0.74), t(40) = -2.508; p = .016.

**Table 3:** Results of power measures.

	Measurement-Points			Test statistics			
	Baseline	After on session	After one week of training	Baseline to after one session		Baseline to after one week of training	
Variable	M (SD) [N]	M (SD) [N]	M (SD) [N]	T (df)	p	T (df)	p
MMG_HK	7.54 (2.71) [41]	-	7.15 (3.29) [41]	-	-	0.94 (40)	.352
MMG_FK	6.15 (2.31) [41]	-	6.93 (3.17) [41]	-	-	-1.92 (40)	.062
Power (Questionnaire)	25.20 (3.68) [41]	25.78 (2.94) [27]	26.51 (3.70) [41]	-1.54 (26)	.135	-3.62 (40)	.001*
VAS_Power	39.78 (19.15) [41]	47.80 (20.09) [41]	42.71 (20.82) [41]	-4.75 (40)	.000*	-0.97 (40)	.336
SAM_Dominance	2.65 (0.74) [40]	2.90 (0.87) [40]	2.50 (0.75) [41]	-2.51 (39)	.016	1.64 (39)	.110

Note. M = Mean. SD = Standard Deviation. T = T-Test-Statistic. p = Significance Level.

Abbreviations and Score Ranges.

* = Statistically significant difference after Holm-Bonferroni adjustment

For the analysis a two-tailed test was applied.

MMG_HK = Multi Motive Grid (Hope for Control) (0–12)

MMG_FK = Fear of Losing Control (0–12)

Power (Questionnaire) = SOPS 6 GV (Sense of Power Scale 6 German Version) (6–36)

VAS_ Power = Visual Analog Scale—Power (0–100)

SAM_Dominance = Self Assessment Manekin Dominance Item (1–5)

Regarding the effect of one week of power posing on individuals' sense of personal power, results showed that there was a significant difference between baseline and a one-week power posing training on the German Sense of Power Scale (SOPS 6 GV) (t(40) = -3.622; p = .001), with participants displaying higher scores after the training (*M* = 26.51; *SD* = 3.70) than before (M = 25.20; SD = 3.68). Regarding the implicit power measure MMG, scores on the 'hope for control' dimension (MMG-HK) were lower after one week of power posing training (M = 7.15; SD = 3.25) than at baseline (M = 7.54; SD = 2.71). However, this difference was not significant (t(40) = 0.942; p = .352). On the MMG 'fear of losing control' dimension (MMG-FK), scores were higher after one week of power posing training (M = 6.93; SD = 3.17) than at baseline (M = 6.15; SD = 2.31), though, this difference was also not significant (t(40) = -1.920; p = .062). For a full list of mean scores and standard deviations for the power measures, please refer to Table 3.

## Discussion

The central aim of the study was to investigate whether power posing had an effect on individuals' interoceptive ability. Firstly, we measured the impact of a single power posing session on interoceptive ability in 41 healthy females. Then, the same participants were randomly assigned to two conditions (daily power posing practice vs. no practice). After one week the conditions alternated. Interoceptive accuracy, measured by the heartbeat tracking task and interoceptive sensibility, measured by the Body Perception Questionnaire (BPQ) as well as confidence ratings, were assessed at baseline, after a single power posing session and after one week of training. The main finding was that power posing significantly increased individuals' interoceptive accuracy after one session of power posing. Power posing reduced interoceptive sensibility, measured by the Body Perception Questionnaire, only after one week of daily practice. In the following paragraphs, the results regarding each interoceptive dimension will be discussed in more depth, together with their clinical implications and future directions.

As expected we found that a single power posing session significantly increased individuals' interoceptive accuracy. This finding is in keeping with previous research, showing that the induction of power appears to foster individuals' perception of bodily signals [34, 43, 44, 74]. Unlike previous studies that used cognitive priming (i.e. a top-down approach) as a mechanism to activate subjective feelings of power [34, 44], we focused on the adoption of powerful physical postures (i.e. a bottom-up approach). The induction of power through an embodiment intervention is in line with theories of grounded cognition, which postulate that the adoption of bodily postures elicits affective states and related cognitions [75]. A possible mechanism behind increases in interoceptive accuracy through power posing could be that the strengthening of subjective feelings of power due to the adoption of a physical pose made it likely for individuals to shift their attention inwards, which offered them more capacity to notice their bodily signals [34, 74]. Additionally, it might be that the adoption of a powerful posture lead to a bodily state associated with relaxation and non-threat (e.g. via a reduction in respiratory rate or physiological arousal) [76–78], which might have fostered sensitivity to heartbeat perception through the absence of stress-related physiological responses. A recent study by Hackford et al. [79], for example, found that the adoption of an upright walking posture was associated with a significantly lower galvanic skin response, lower systolic blood pressure response, and marginally lower skin temperature compared to the adoption of a slumped walking posture. Also, Schmid and colleagues [77] showed that individuals who were primed with power showed significantly lower physiological arousal (i.e. lower heart rate increase) after a social stress test compared to controls. On the other hand, there is also research suggesting that powerful postures can lead to increases in physiological arousal [80], reflecting the activation of the behavioural approach system [81]. In this regard, Scheepers and colleagues [82] showed that priming individuals with high power was associated with an efficient cardiovascular pattern ('challenge'), reflected in high cardiac performance (cardiac output), coupled with low total peripheral resistance. As high cardiac outflow, in turn, has been associated with increased interoceptive accuracy [83, 84], it could be that the cardiovascular pattern induced by the power posing might have fostered performance on the HBTT. Of course, these possible explanations are hypothetical and warrant further investigation.

Using powerful postures as a way to induce power might be particularly beneficial as Huang and colleagues [47] showed that bodily postures had a higher impact on outcomes associated with power, than associative priming. However, it is important to note that we did not find evidence that one week of power posing training maintained the initial increase in interoceptive accuracy. There are several reasons that could explain this unexpected finding. Firstly, it could be that the adoption of a powerful posture has an immediate, temporary effect that decreases when the posture is no longer held. This idea would be in keeping with dynamically changing bodily states as a consequence of physical and context-dependent alterations [85–87]. Alternatively, it may also be that one week of training is simply not enough time to lead to a maintained increased effect. Some studies on the malleability of interoceptive accuracy have shown that longer periods of sustained practice are required to achieve lasting change. Bourneman and Singer [88], for example, found that significant improvements in interoceptive accuracy could only be reached after six months of contemplative practice. It would, therefore, be beneficial to investigate whether effects can be found when power posing is practiced for several weeks.

Regarding interoceptive sensibility, unlike expected, power posing did not significantly improve individuals' confidence in their performance on the HBTT task after a single session or one week of power posing. From a theoretical point of view, power posing could potentially increase individuals' confidence in subjective performance, as confidence in one's performance has been associated with the induction of power [89–91]. However, performance confidence has also been shown to represent a trait-like construct, which appears to remain stable across different tasks [92, 93]. Thus, here as well, one week of power posing might not be a sufficient amount of time to alter confidence in performance. In line with this assumption, Parkin and colleagues [94] found no changes in interoceptive confidence after one week of mindfulness practice. They did, however, report a significant increase in confidence rating scores after eight weeks of training. Similarly, Fischer et al. [33] reported no significant increase in confidence scores after four weeks of body scan training, they did, however, find significant improvements, after eight weeks of practice. It is further important to note, that power posing targeted the manipulation of individuals' *self-focus* and heightened self-focus might not directly relate to increases in performance confidence [95, 96]. Finally, the finding that the power manipulation did not affect confidence, but did affect interoceptive accuracy in the short-term, provides support for the previously proposed independence between interoceptive accuracy and sensibility (measured by confidence) [1, 60, 97]. This assumption is also supported by the finding that no significant correlations between interoceptive accuracy and confidence were found at baseline, after one session of power posing, after one week of daily practice or after one week of practice pause.

When assessing interoceptive sensibility using the BPQ awareness subscale, a significant reduction in BPQ scores was observed after one week of daily power posing practice. As a significant increase was not observed for interoceptive accuracy or confidence at this time point, this observation further underlines the assumption that the different dimensions of interoception appear to diverge from one another. As there is still very little evidence regarding the association between interoceptive sensibility (measured by BPQ) and other cognitive- and emotional constructs, the implications that can be drawn from of our observation remain limited. However, one aspect that could be important to consider in regards to this finding is that although the BPQ is commonly applied as a measure assessing interoceptive sensibility, it does not distinguish between adaptive and maladaptive bodily awareness [98]. The majority of the items appear to reflect subjective sensations that seem to be bothersome to the individual [98]. Based on this observation, it can be speculated that the power manipulation might have affected a dimension of interoceptive sensibility that reflects subjective sensitivity to unpleasant bodily signals. To clarify this issue, it would be valuable to integrate other self-report questionnaires assessing interoceptive sensibility, such as the Multidimensional Assessment of Interoceptive Awareness (MAIA) [98], in the future. The MAIA has the advantage that it not only assesses the 'noticing' dimension of interoceptive sensibility, but several others, such as the ability to sustain attention to bodily sensations or the tendency not to react with emotional stress to bodily sensation [98]. Also, it investigates responses to pleasant, unpleasant and neutral bodily sensations, thus, discriminates between adaptive and maladaptive aspects of interoceptive sensitivity [99]. Using this additional measure in future research could help to get a better understanding of the specific facets of interoceptive sensibility power posing could potentially influence.

Considering the findings on interoceptive awareness, we found a significant short-term effect of power posing on interoceptive awareness, with a decline in interoceptive awareness appearing after a single power posing session. This finding is logical because interoceptive awareness reflects the concordance between interoceptive accuracy and interoceptive sensibility measures [1]. As interoceptive accuracy improved and confidence rating remained stable, the concordance between the two different dimensions was reduced, meaning that individuals did not integrate their increase in objective performance into their subjective belief about their performance. This failure to optimally incorporate ‘bottom-up’ interoceptive signals when updating ‘top-down’ subjective beliefs is considered an *interoceptive prediction error* [100]. The question is, what impact this observed divergence (i.e. a state change in interoceptive awareness) has on other domains of functioning such as, for example, emotional experience. To this date, no previous studies looking at the effects of improved interoceptive accuracy have addressed the potential modulating role of interoceptive sensibility [101]. On the one hand, as interoceptive accuracy and sensibility are considered independent of one another [1], effects that may emerge as a result of enhanced interoceptive accuracy in domains (such as the experience or regulation of emotions), are likely to occur even when individuals do not subjectively believe themselves to have better interoceptive ability. However, on the other hand, previous research has found that *trait* interoceptive prediction errors correlated with lower emotional sensitivity, the occurrence of anxiety symptoms [100], abnormal skin sensations [102] and emotional eating [103]. Thus, a large discrepancy between the two dimensions may be disadvantageous for the individual. In this pilot study we focused on the effects of power posing on *state* interoceptive awareness and did not relate the observed changes to secondary outcomes. It would, therefore, be interesting to investigate in the future how the awareness dimension of interoception relates to other aspects such as e.g. anxiety and stress, when power posing is practiced over a longer time period. [101].

Regarding the results of the power measures, individuals showed increased VAS scores after a single power posing session and a heightened sense of personal power after one week of training, measured by questionnaire. Both findings support our third hypothesis, by indicating that individuals experienced themselves as more powerful after power posing than before. This observation converges with the literature on power posing, with the majority of studies reporting a heightened subjective sense of power after power posing [46, 104]. These findings are important as *'felt power'* has been associated with an increase in positive- [80, 105] and a reduction in negative affect [106]. It might, therefore, be beneficial to test the long-term application of power posing in clinical samples, which display particular deficits in this domain.

Regarding practical implications, the current study supports that power posing seems to have an immediate, temporary effect on interoceptive accuracy, as well as a one-session and a training effect on subjective feelings of power. As power posing seems to 'boost' the perception of bodily signals, it could be particularly beneficial to apply in situations where sensitivity to self-relevant physiological information is required (e.g. intuitive judgment and decision making [4], emotional processing [8] or body image [13]). Also, it might be advantageous as a one-session intervention for individuals who display low IAcc and reduced subjective feelings of power, for example, those with anorexia nervosa [21, 107] or depression [18, 108]. The benefit of training power posing daily for one week to increase interoceptive accuracy is not supported by our findings. Also, our study did not find that it leads to improvements in individual’s confidence in their performance on the HBTT. However, one week of power posing practice seems to reduce the perception of bodily sensations that are bothersome to the individual (measured by questionnaire). Therefore, practicing power posing might also be beneficial for individuals displaying high scores on the BPQ, for example, patients with joint hypermobility [109], generalized anxiety- or panic disorder [110].

The study has several limitations. Firstly, holding a particular posture for a certain amount of time always goes hand in hand with some kind of self-focus, as it requires moment-to-moment self-monitoring to keep the body correctly adjusted. Therefore, it is very difficult to disentangle the effect of the power postures from the effect of self-focus that might have resulted from holding the poses. To clarify this question one could integrate neutral postures, as well as postures expressing low power in a future study and compare these with powerful postures. Theoretically, the powerful postures should lead to better interoceptive accuracy than the neutral or powerless postures, as high power seems to foster mechanisms that initiate and maintain self-focus more than low power. Regarding the limitations of the study further, the Schandry task has been criticised for being susceptible to non-interoceptive influences, such as prior knowledge about heart rate, guessing one's heart rate, time estimations and practice effects (for further information, please see [111–114]) and we did not control for these possible confounding factors. One reason for this was that assessing beliefs about their heart rate prior to task completion has been proposed to influence performance on the task [115]. One strategy to bypass this possibility could have been to ask individuals about it after the task had been completed for the first time. However, as we tested the subjects again at other time points, the question about their heart rate knowledge might have influenced their subsequent responses. Regarding the influence of time estimates, several recent studies that included a time estimation task, did not find a positive relationship between time estimates and interoceptive accuracy, and controlling for time estimates did not change the relationship between interceptive accuracy and other outcomes [116, 117]. It is therefore unlikely that time estimates confounded the results, especially as interoceptive accuracy did not significantly increase after one week of training in our study. However, it would be beneficial to replicate the study and control for these factors to confidently exclude them as confounding variables. Furthermore, we do not know whether the participants engaged in any form of uncontrolled practice of interoceptive enhancement during the week of practice or pause, such as counting their own heartbeats. However, we did inquire whether they practiced any other body-centered intervention during the week of practice or pause (e.g. yoga), which none of the participants confirmed. Finally, we used a female sample only, thus the results are not generalizable to men.

Regarding future directions, it would be valuable to investigate the effect of power posing on interoceptive ability in clinical populations, for example, individuals with anorexia nervosa, who have been shown to display deficits in interoceptive accuracy throughout the course of therapy [118] and have been reported to perceive themselves as powerless [107, 119]. Also, it would be important to explore how changes that can be observed in the different interoceptive dimensions due to specific interventions relate to improvements in other domains of functioning such as emotional experience, behavioural self-regulation and quality of life, to get a better understanding of the areas that could be targeted with practices that have been found to improve interoceptive abilities.

## Conclusion

Our study was the first to investigate the effects of adopting powerful postures on the different dimensions of interoceptive ability. We conclude that power posing has the potential to improve interoceptive accuracy in the short-term and reduce scores on the BPQ awareness subscale after one week of practicing. Further research should investigate, whether the same effect can be found in clinical populations, which display particular deficits in interoceptive ability.

## Supporting information

S1 File Sense of power scale—6—English version. (PDF)

S2 File Sense of power scale—6—German version (SOPS 6 GV). (PDF)

S1 Table Overview measures. (PDF)
