# Supplementary material for: Improving interoceptive ability through the practice of power posing: A pilot study
Source: PLoS One. 2019 Feb 7;14(2):e0211453. doi: 10.1371/journal.pone.0211453 (PMC6366763; doi:10.1371/journal.pone.0211453)
Supplement: S1 File — (PDF) [file pone.0211453.s001.pdf]

Sense of Power Scale 6 (Englisch Version) (SOPS E 6)

In rating each of the items below, please use the following scale:

1	2	3	4	5	6
Disagree strongly	Disagree	Disagree a little	Agree a little	Agree	Agree strongly

______ 1. I can get him/her/them to listen to what I say.

______ 2. My wishes do not carry much weight. (r)

______ 3. I can get him/her/them to do what I want.

______ 4. Even if I voice them, my views have little sway. (r)

______ 5. I think I have a great deal of power

______ 6. My ideas and opinions are often ignored (r)

Please note that this scale was translated into German for the presented study. The original *Sense of Power Scale* was constructed and published by Anderson, John & Keltner (2012). Permission to use the items of the scale was obtained before the study commenced. We only used the first six items of their original scale and our participants could rate the items on a scale from 1-6.
