# Supplementary material for: Improving interoceptive ability through the practice of power posing: A pilot study
Source: PLoS One. 2019 Feb 7;14(2):e0211453. doi: 10.1371/journal.pone.0211453 (PMC6366763; doi:10.1371/journal.pone.0211453)
Supplement: S2 File — (PDF) [file pone.0211453.s002.pdf]

Skala zur Erfassung der subjektiven Empfindung von Macht
Sense of Power Scale 6 German Version (SOPS 6 GV)

Bitte verwenden Sie die folgende Skala zur Beantwortung der unten aufgeführten Aussagen. Die Aussagen beziehen sich auf Ihre Beziehung zu Anderen. In wie weit stimmen Sie den Aussagen zu bzw. nicht zu?

1	2	3	4	5	6
Ich stimme ganz und gar nicht zu	ich stimme nicht zu	ich stimme eher nicht zu	ich stimme eher zu	ich stimme zu	ich stimme voll und ganz zu

______ 1. Ich kann andere dazu bewegen mir zuzuhören

______ 2. Meine Wünsche haben nicht viel Einfluss bei anderen (r)

______ 3. Ich kann andere dazu bewegen das zu tun, was ich will

______ 4. Selbst wenn ich mich klar ausdrücke, haben meine Aussagen wenig Überzeugungskraft (r)

______ 5. Ich denke, dass ich sehr viel Macht habe

______ 6. Meine Ideen und Meinungen werden oft ignoriert (r)

Die Skala wurde aus dem Englischen übersetzt. Die Original-Skala wurde von Anderson, John & Keltner (2012) erstellt. Es wurden nur die ersten sechs Items der Original-Skala verwendet und unsere ProbandInnen konnten diese auf einer Skala von 1-6 bewerten. Die Genehmigung zur Verwendung der Original-Items der Skala in dieser Studie liegt vor.
