# Supplementary material for: Improving interoceptive ability through the practice of power posing: A pilot study
Source: PLoS One. 2019 Feb 7;14(2):e0211453. doi: 10.1371/journal.pone.0211453 (PMC6366763; doi:10.1371/journal.pone.0211453)
Supplement: S1 Table — (PDF) [file pone.0211453.s003.pdf]

Time Point	Measurement	Intervention
**One week prior to testing (online)**	Demographics Questionnaire (including age, education, level of fitness, previous experience with body centred interventions); State Trait Anxiety Inventory - Trait Scale (Spielberger et al., 1970); Patient Health Questionnaire (PHQ) (Löwe, 2001)	
**Baseline (T0)**	Hand Grip Task (HGT) (as part of the cover story); Multi Motive Grid (MMG) (Sokolowski et al., 2000); Self-Assessment Manekin Scale (SAM Bradley & Lang, 1994); Visual analogue scale (VAS) - Power (created by the authors); Sense of Power Scale 6 German Version (SOPS 6 GV) (created by the authors); Body Perception Questionnaire (BPQ) (Porges, 1993); Heartbeat Detection Task (Schandry, 1981); Subjective confidence rating (Garfinkel et al., 2015)	
		Explanation of power poses and short practice
		Intervention of adopting the three power poses
**After single power posing session (T1)**	Heartbeat Detection Task and subjective confidence rating; HGT; SAM; VAS; SOPS-6-GV; BPQ	
**After T1 was completed**	*Randomisation of all participants into two groups (Group A and Group B)*	
**Between T1 and T2**	Daily Diary (Group A) (created by the authors)<br>No Diary (Group B)	Group A: practised power posing twice daily<br>Group B: practised not at all
**After one week of practice or no practice (T2)**	HGT; MMG; SAM; VAS; SOPS-6-GV; BPQ; Heartbeat Detection Task and subjective confidence rating	
**Between T2 and T3**	No Diary (Group A)<br>Diary (Group B)	Group A: practised not at all<br>Group B: practised power posing twice daily
**After one week of practice or no practice (T3)**	HGT; MMG; SAM; VAS; SOPS-6-GV; BPQ; Heartbeat Detection Task and subjective confidence rating	
